# Supplementary material for: Mitotic Dysregulation at Tumor Initiation Creates a Therapeutic Vulnerability to Combination Anti-Mitotic and Pro-Apoptotic Agents for MYCN-Driven Neuroblastoma
Source: Int J Mol Sci. 2023 Oct 25;24(21):15571. doi: 10.3390/ijms242115571 (PMC10649872; doi:10.3390/ijms242115571)
Supplement: Supplementary file 1 [file ijms-24-15571-s001.zip › mitosis Supp Figures_all.pdf]

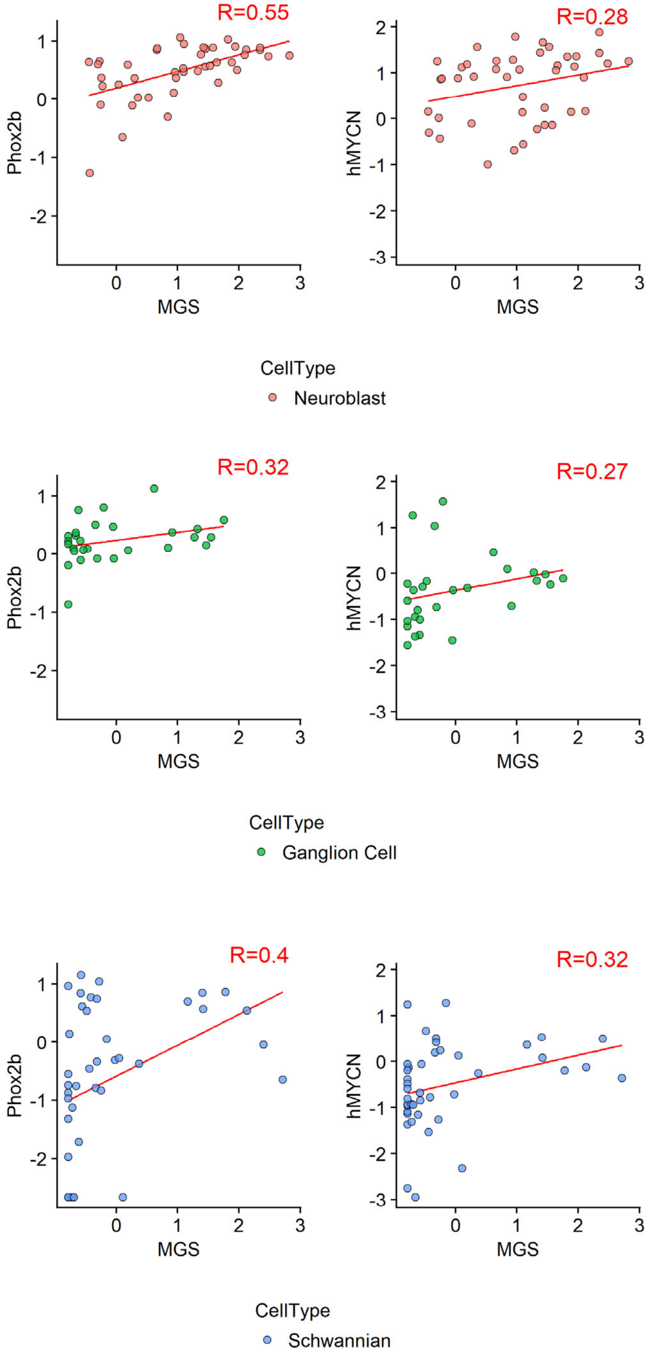

Supplementary Figure S1: Correlation comparing MGS and neuroblast markers Phox2b (left) and hMYCN (right) for expression single cells from TH-MYCN+/+ ganglia and tumors. R value refers to Pearson correlation statistic. Color corresponds to cell type groupings.

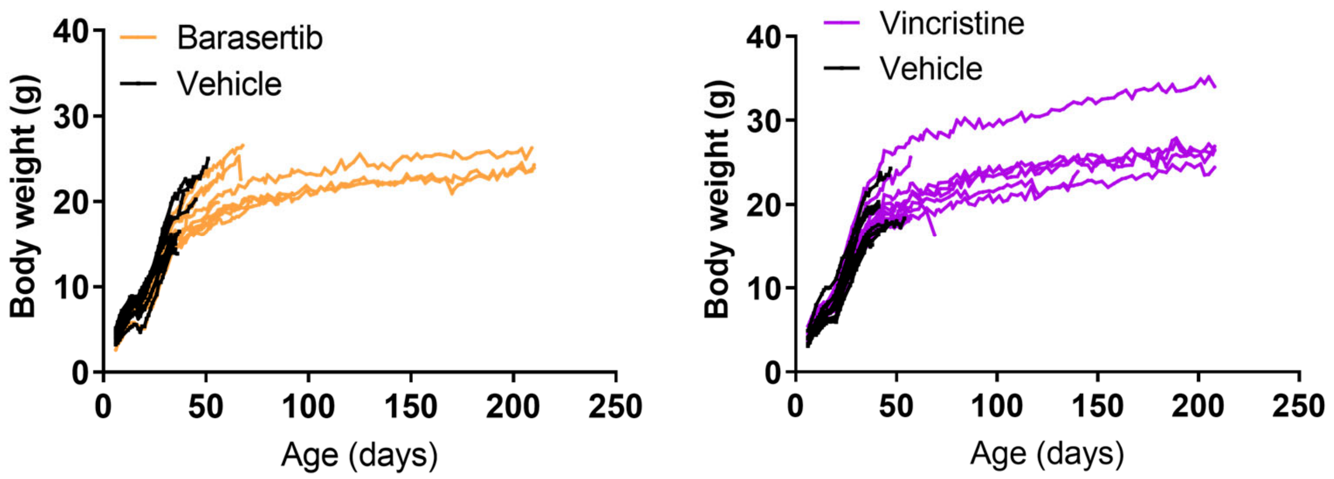

Supplementary Figure S2. Prophylactic treatment using antimitotic compounds at low dosage does not affect normal weight development. Data represents body weight of individual TH-MYCN +/+ mouse treated with barasertib, vincristine or vehicles 4 days per week, from 6 day-old.

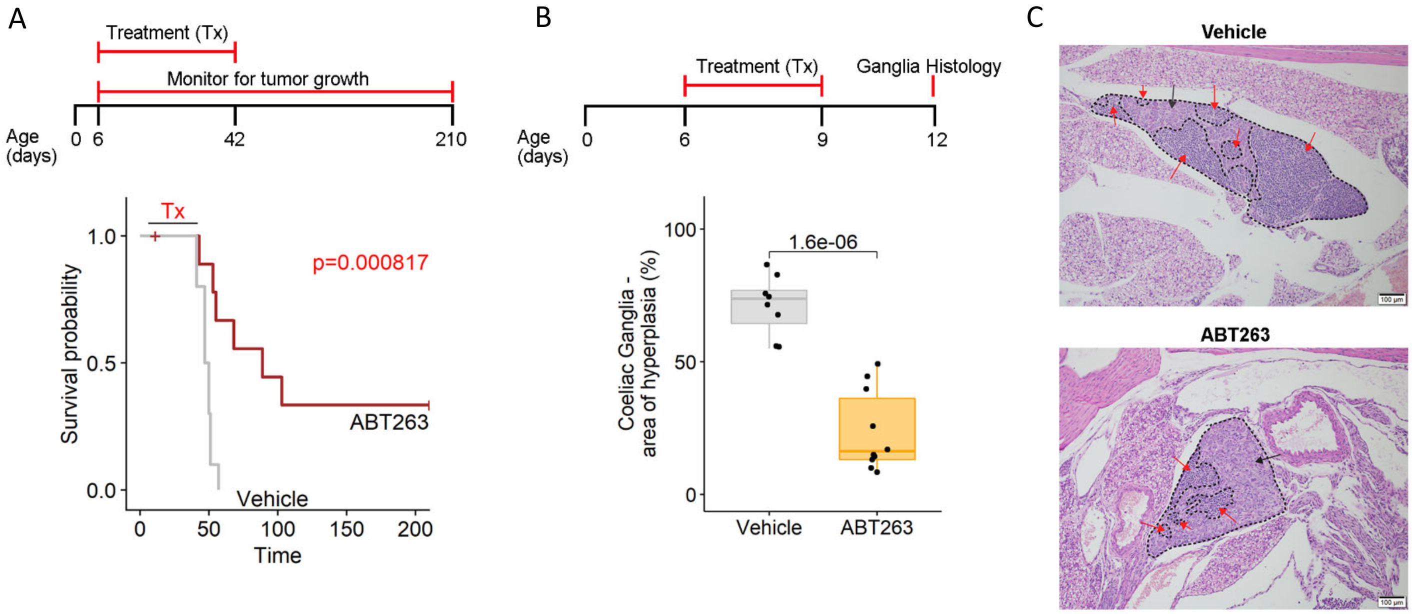

Supplementary Figure S3. A) Kaplan Meier curve for TH-MYCN+/+ mice survival assessing vehicle vs 100mg/kg/day ABT263. Treatment was from day 6 until day 42 on a 4 days on/ 3 days off schedule for a total of 22 doses. Endpoint was considered to be time until maximum palpable tumor detected (10mm diameter) or 210 days, whichever came first. P-value comparing treatments was calculated using log-rank tests. B) Boxplot comparing different treatment groups for percentage of hyperplasia in the coeliac ganglia of treated mice: vehicle vs 100mg/kg/day ABT263. Treatment was from day 6 until day 9 for a total of 4 doses. Ganglia were detected in hematoxylin and eosin sections of mice at 12 days of age. C) Representative hematoxylin and eosin sections from Figure 6B showing hyperplastic (red arrows) and non-hyperplastic (black arrows) regions in coeliac ganglia.

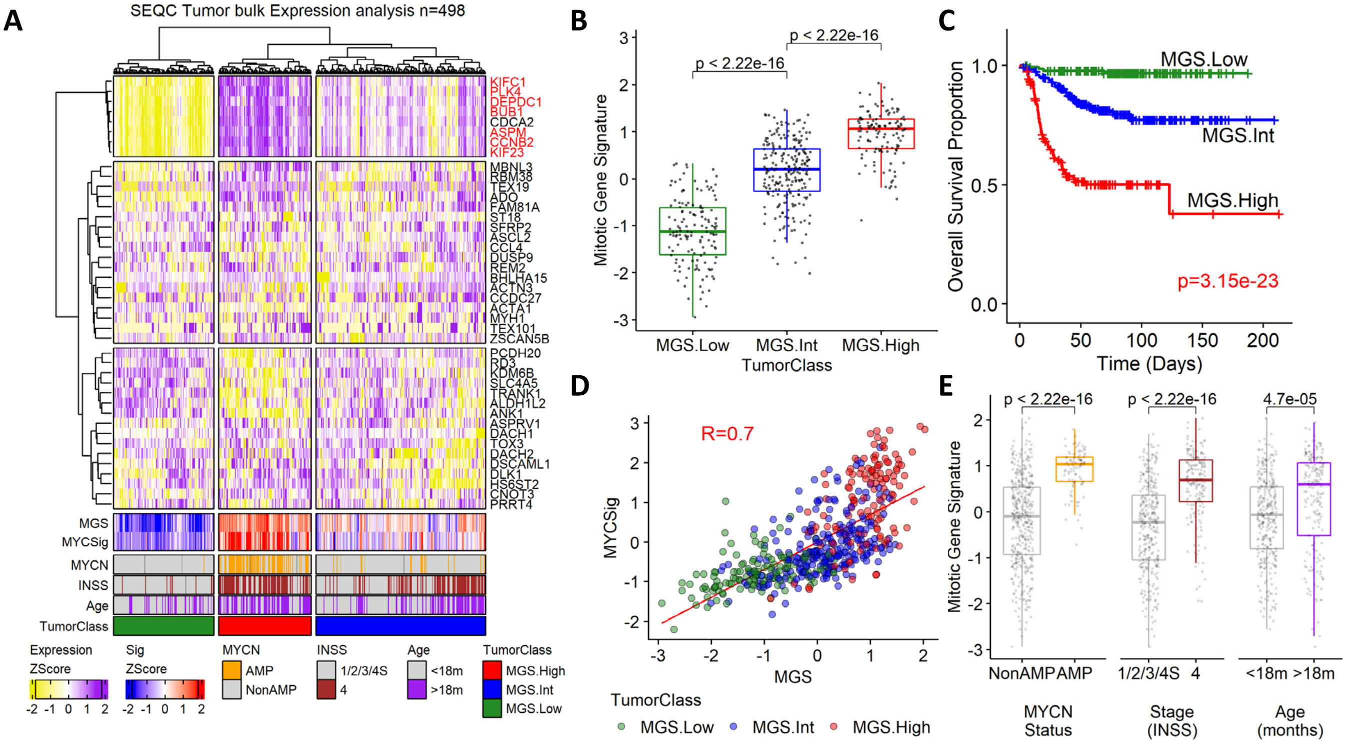

Supplementary Figure S4: A) Heatmap from neuroblastoma RNA-seq[22] for the same genes identified in Figure 1A. Ward.d2 based hierarchical clustering identified three tumor groups (see TumorClass). Red print genes indicate human genes that constitute the MGS. B) Boxplot showing MGS expression in tumor microarray split by tumor grouping in Figure 3A. P-values derived from Wilcoxon rank sum test. C) Kaplan Meier plot for overall survival, split based on tumor groupings identified for Supplementary Figure 3A-B. P-value comparing treatments was calculated using log-rank tests. D) Correlation comparing MGS and a MYC-target gene signature[16] expression in neuroblastoma tumors. R value refers to Pearson correlation statistic. Color corresponds to tumor groupings. E) Boxplot showing MGS expression in neuroblastoma tumors split by clinical groups for MYCN status, Stage and Age. P-values derived from Wilcoxon rank sum test.

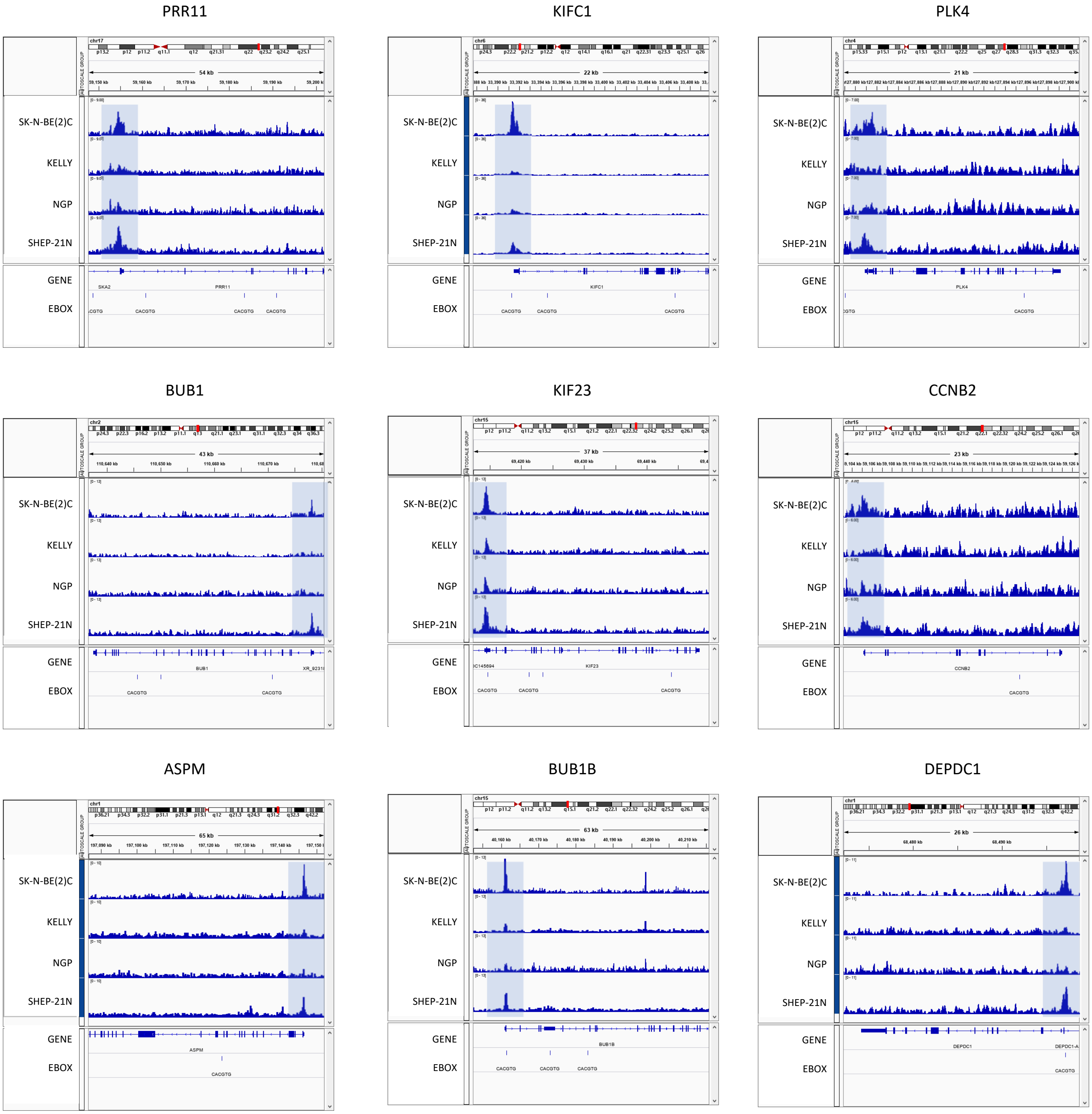

Supplementary Figure S5: MYCN ChIP-sequencing gene tracks for MGS gene regions in neuroblastoma cell lines. Blue shading shows regions of MYCN peaks.

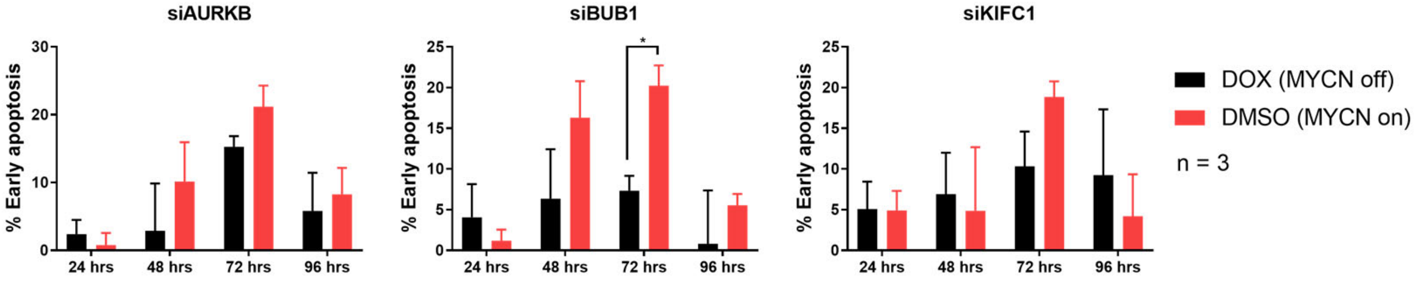

Supplementary Figure S6. Apoptosis and necrosis were measured at 24 - 96 hours post transfection by apoptosis assay using 7-AAD and PE-conjugated Annexin V in Doxycycline-induced (2ug/mL 72 hrs) SHEP.tet21N cells transfected with AURKB, BUB1 and KIFC1 SMARTpool siRNA, and normalised using on-target plus control siRNA transfected cells. Data represents relative change in Annexin V positive, 7-AAD negative staining cells ± standard error from 3 independent biological replicates. Unpaired student t test. *, $P < 0.05$.

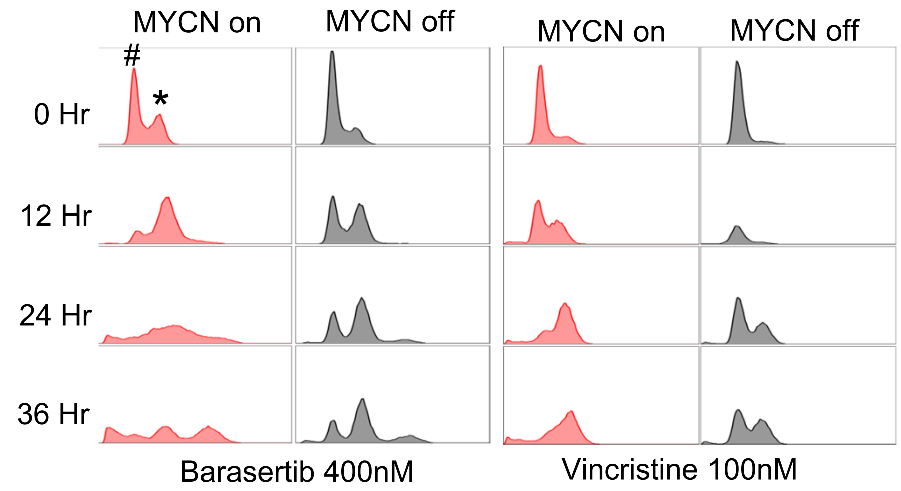

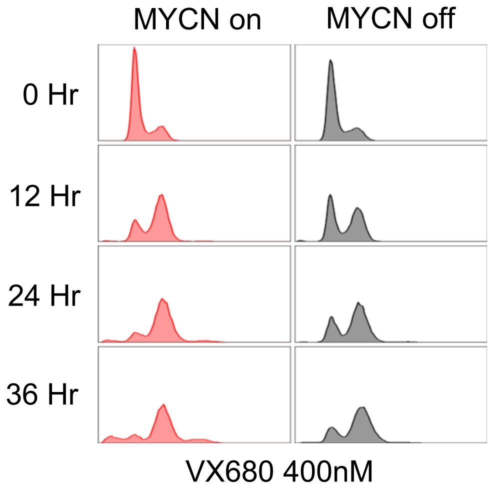

Supplementary Figure S7A. DMSO or doxycycline-induced SHEP.tet21N was treated with antimitotic compounds (vincristine, Barasertib, VX680 or SR31527) for 12 - 36 hours and stained with propidium iodide for cell cycle analysis. Data represents histogram of fluorescence at 670nM. # indicates G1/0, and * indicates G2/M in cell cycle.

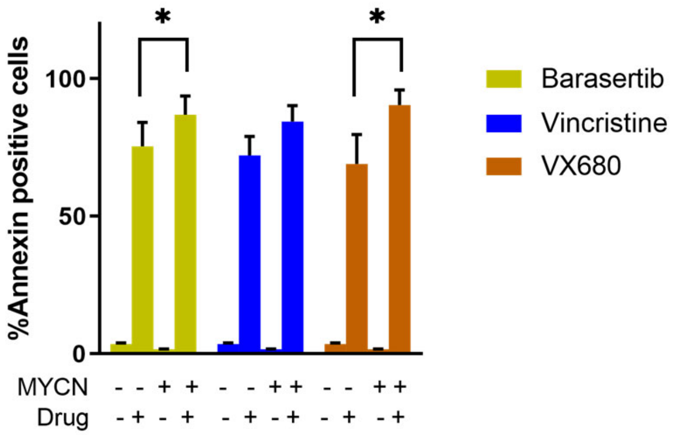

Supplementary Figure S7B. Apoptotic population of DMSO or doxycycline-induced SHEP.tet21N, treated with antimitotic compounds (barasertib, vincristine, VX680) and stained with PE conjugated annexin-V and 7-Aminoactinomycin D for flow cytometry. Data represents averaged annexin positive cell population± standard error from 3 independent biological replicates. Unpaired student t test. *, $P < 0.05$.
